# Usefulness of duckbill‐type anti‐reflux self‐expandable metal stents for distal malignant biliary obstruction with duodenal invasion: A pilot study

**DOI:** 10.1002/deo2.103

**Published:** 2022-03-09

**Authors:** Ikuhiro Kobori, Yasumi Katayama, Fuki Hayakawa, Takeshi Fujiwara, Masaru Kuwada, Yoshinori Gyotoku, Akihiro Kitahama, Yumi Kusano, Masaya Tamano

**Affiliations:** ^1^ Department of Gastroenterology Dokkyo Medical University Saitama Medical Center Saitama Japan; ^2^ Endoscopy Center, Dokkyo Medical University Saitama Medical Center Saitama Japan

**Keywords:** cholangiopancreatography, common bile duct, endoscopic retrograde, self‐expandable metallic stents

## Abstract

**Objectives:**

Early obstruction of a self‐expandable metal stent placed for distal malignant biliary obstruction is more likely to occur in the presence of duodenal invasion. An anti‐reflux self‐expandable metal stent (ARMS) has been developed for the purpose of preventing duodenal fluid reflux into the bile duct. In this study, we evaluated the usefulness and safety of a duckbill‐type ARMS (D‐ARMS) in the situation of duodenal invasion.

**Methods:**

We retrospectively analyzed 10 consecutive patients who received D‐ARMS for distal malignant biliary obstruction with duodenal invasion. We evaluated non‐occlusion cholangitis, recurrent biliary obstruction (RBO), and adverse events after D‐ARMS placement.

**Results:**

There were no cases of non‐occlusion cholangitis. RBO was observed in 2 patients (20%), and time to RBO was 236 days and 117 days, respectively. The causes of RBO were overgrowth and sludge formation. The median time to RBO was 382 days (range, 117–382 days). Only one adverse event was observed (cholecystitis).

**Conclusions:**

D‐ARMS shows potential as an optimal ARMS.

## INTRODUCTION

The various types of biliary drainage for distal malignant biliary obstruction include transpapillary drainage by endoscopic retrograde cholangiopancreatography (ERCP) procedure, percutaneous drainage, and endoscopic ultrasonography (EUS)‐guided drainage. Transpapillary drainage by ERCP is a common procedure if the endoscope can reach the papilla, and is performed at many facilities. The usefulness of biliary drainage with a self‐expanding metal stent (SEMS) is widely recognized, but sludge formation and food impaction are cited as causes of stent obstruction.^1^, ^2^, ^3^ Sludge formation and food impaction are associated with the reflux of duodenal fluid into the bile duct,^4^, ^5^, ^6^, ^7^, ^8^ and the presence of duodenal invasion causes hypoperistalsis, making duodenal fluid more likely to reflux into the bile duct. Therefore, early obstruction of SEMS is more likely to occur in the presence of duodenal invasion.^9^, ^10^, ^11^, ^12^, ^13^, ^14^ The anti‐reflux self‐expandable metal stent (ARMS) has been previously described for the purpose of preventing duodenal fluid reflux.^15^, ^16^, ^17^, ^18^, ^19^, ^20^, ^21^, ^22^ In this study, we examined the usefulness and safety of a duckbill‐type anti‐reflux self‐expandable metal stent (D‐ARMS; Kawasumi Laboratories, Tokyo, Japan) for use in the presence of duodenal invasion.

## MATERIALS AND METHODS

### Study design

We conducted a retrospective study of consecutive patients who received a D‐ARMS for distal malignant biliary obstruction with duodenal invasion at the Dokkyo Medical University Saitama Medical Center, Japan. The study was approved by the review board at Dokkyo Medical University Saitama Medical Center (approval no. 21036, approval date 4 June 2021) and was conducted in accordance with the tenets of the Helsinki Declaration. Evaluation of D‐ARMS was based on the TOKYO criteria 2014.^23^ The primary outcome was the rate of non‐occlusion cholangitis after D‐ARMS placement. The secondary outcomes were the cause of recurrent biliary obstruction (RBO), time to RBO (TRBO), technical success, functional success, and adverse events. RBO was defined as the recurrence of obstructive jaundice and/or cholangitis due to stent occlusion or migration. TRBO was defined as the length of time between stent placement and the occurrence of RBO. Technical success was defined as the successful deployment of a D‐ARMS in the intended location with sufficient coverage of the stricture. Functional success was defined as (1) a 50% decrease in or normalization of the bilirubin level within 14 days of stent placement; and (2) in the case of an unoccluded stent replaced with a D‐ARMS, no exacerbation of the bilirubin level within 14 days of D‐ARMS placement.

### Patients

Ten consecutive patients with distal malignant biliary obstruction and duodenal invasion were treated with D‐ARMS between December 2019 and March 2021. Distal malignant biliary obstruction was diagnosed based on the clinical, laboratory, radiological, and pathological findings. Distal malignant biliary obstruction was defined as a site of stenosis at least 2 cm away from the liver hilum. Malignancy was diagnosed pathologically by EUS‐guided fine needle aspiration or ERCP. The duodenal invasion was diagnosed when duodenal erosions, ulcers, or strictures thought to have been caused by malignancy were observed endoscopically or when invasion into the duodenal muscular layer was confirmed by EUS, regardless of whether these findings were confirmed pathologically. Patients with resectable tumors or surgically altered anatomy were excluded. Patients who underwent biliary drainage, as well as initial drainage, were included.

### Stent design

**FIGURE 1:**
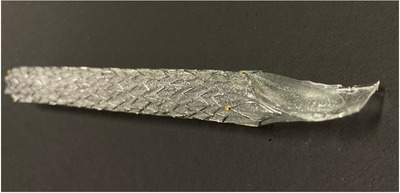
The duckbill‐type anti‐reflux self‐expandable metal stent

The D‐ARMS is a laser‐cut covered SEMS with a 12.5 mm duckbill‐shaped anti‐reflux valve attached to the duodenal end (Figure 1). It is made of nitinol wire, and an expanded polytetrafluoroethylene membrane covers the stent to form a valve structure. The valve is normally closed to prevent the backflow of duodenal fluid into the bile duct but opens to allow bile to flow when the pressure in the bile duct increases. The diameter of the delivery system was 9 Fr and the shortening rate was 4%. In this study, we used D‐ARMS with 10 mm diameter and 60 mm length.

### Procedures

**FIGURE 2:**
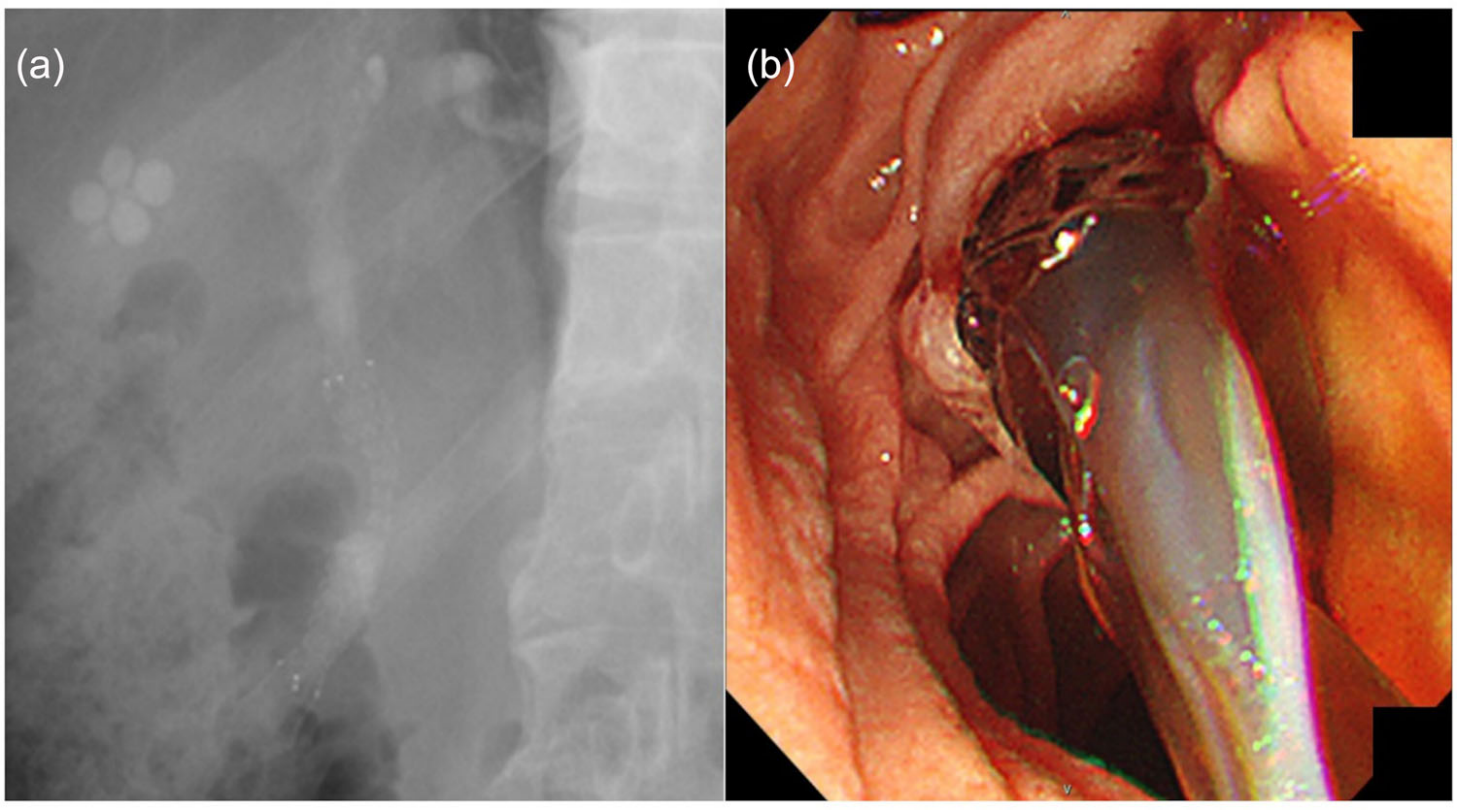
Fluoroscopic (a) and endoscopic (b) images showing duckbill‐type anti‐reflux self‐expandable metal stent (D‐ARMS) placement. Abbreviations: D‐ARMS, duckbill‐type anti‐reflux self‐expandable metal stent

D‐ARMS placement was performed by a normal ERCP procedure using a standard side‐viewing duodenoscope (JF‐260V or TJF‐260V; Olympus Medical, Tokyo, Japan). Endoscopic sphincterotomy was performed in all patients. D‐ARMS length was determined by the cholangiographic findings and was 60 mm in all patients. Patients who had previously undergone biliary drainage received D‐ARMS placement immediately after removing the previous stent. In the case of concurrent cholangitis, balloon sweeping was performed when biliary sludge or food residue was suspected. The stent was placed across the papilla so that the end of the stent on the papillary side protruded 5–10 mm into the duodenum (Figure 2).

### Follow‐up

The patients were followed up at our hospital or a transferred hospital until death or until 31 July 2021, whichever came first. Computed tomography was performed when adverse events were suspected based on the clinical symptoms or blood tests. Adverse events other than recurrent biliary obstruction were categorized as follows: pancreatitis, non‐occlusion cholangitis, cholecystitis, and others (bleeding, ulceration, penetration, perforation, aspiration pneumonia); and were categorized as early (within 30 days) or late (31 days or later). The time point of adverse events was defined as the point when symptoms associated with these conditions were observed.

### Statistical analyses

Statistical analyses were performed using JMP version 14 software (SAS Institute, Cary, NC, USA). TRBO was estimated using the Kaplan–Meier method.

## RESULTS

### Patient characteristics

The patient characteristics are summarized in Table 1. The median age was 73.5 (range, 53–85) years and there were six males and four females. The causes of malignant biliary obstruction were pancreatic cancer (*n* = 7, 70%), bile duct cancer (*n* = 1, 10%), and ampullary cancer (*n* = 2, 20%). D‐ARMS was the first stent placement in one patient (10%), a plastic stent had been placed previously in five patients (50%), a covered SEMS had been placed previously in three patients (30%), and a percutaneous transhepatic biliary drainage tube had been placed previously in one patient (10%). One patient was placed with D‐ARMS as the initial drainage because he had obvious duodenal invasion and multiple liver metastases, and was diagnosed as unresectable at the time of ERCP. Of the three patients with a covered SEMS, two patients had a D‐ARMS placed after SEMS removal, and one patient had a stent‐in‐stent placement that added a D‐ARMS inside a SEMS. Chemotherapy was performed in seven patients (70%) after D‐ARMS placement. The mucosal invasion was confirmed endoscopically in nine patients (90%) and invasion into the duodenal muscular layer was confirmed by EUS in one patient (10%). Tumor invasion to the duodenum was categorized according to the Mutignani classification as type I (*n* = 7), type II (*n* = 2), and type III (*n* = 1).^24^ Therapeutic intervention for duodenal tumor invasion was performed when gastric outlet obstruction symptoms were observed: duodenal stent placement was performed in one patient and gastrojejunal bypass surgery was performed in one patient, with each performed after D‐ARMS placement. The duodenal stent placement was performed 68 days after the D‐ARMS placement, and the Mutignani classification was type I. The gastrojejunal bypass surgery was performed 56 days after D‐ARMS placement, and the Mutignani classification was type III.

**TABLE 1:** Patient characteristics

Characteristic	Value
Age (years)	73.5 (53–85)
Sex (male/female)	6/4 (60/40)
Primary cancer	
Pancreatic cancer	7 (70)
Bile duct cancer	1 (10)
Ampullary cancer	2 (20)
Length of D‐ARMS	
60 mm	10 (100)
Chemotherapy after D‐ARMS placement	7 (70)
Prior drainage	9 (90)
Plastic stent	5 (50)
Covered self‐expandable metal stent	3 (30)
Percutaneous transhepatic biliary drainage	1 (10)
Duodenal invasion site †	
Type I	7 (70)
Type II	2 (20)
Type III	1 (10)
Therapeutic intervention for duodenal invasion	2 (20)
Duodenal stent	1 (10)
Gastrojejunal bypass surgery	1 (10)

*n* = 10. Data are expressed as median (range) or number (%).

^＊^Continuous variables are presented as medians (ranges), categorical variables as absolute numbers (percentages).

^†^Tumor invasion to the duodenum was categorized according to the Mutignani classification.^24^

Abbreviation: D‐ARMS, duckbill‐type anti‐reflux self‐expandable metal stent.

### Outcomes

**FIGURE 3:**
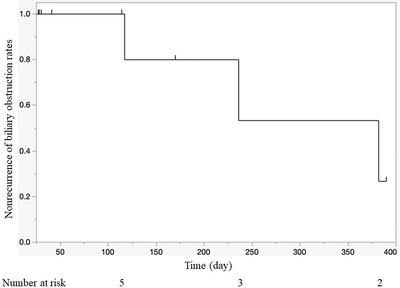
Kaplan–Meier curve of time to recurrent biliary obstruction. The small vertical bars indicate censored cases

Table 2 summarizes the clinical outcomes of D‐ARMS. Both technical and functional success was achieved in all patients (100%). The median duration of observation was 133 days (range, 27–434 days), during which eight patients (80%) died due to progression of primary cancer. RBO was observed in two patients, in whom TRBO was 236 days and 117 days. In both of these patients, SEMS was placed before D‐ARMS placement, and the causes of RBO were overgrowth and sludge formation. It was difficult to remove the D‐ARMS when re‐intervention was performed. An endoscopist with experience of more than 500 ERCP cases performed all attempted removals. Despite sufficient working space when performing ERCP, removal was difficult due to resistance when using snare forceps. In the case of overgrowth, the anti‐reflux valve remained, and a plastic stent was additionally placed through the mesh gaps in the D‐ARMS. As the anti‐reflux valve was collapsed in the case of sludge formation, an additional SEMS was placed after cleaning the bile duct. In the analysis by duodenal invasion site, RBO developed in two of seven cases of type I, and RBO was not observed in types II or III within the observation period. Median TRBO was 382 days (range, 117–382 days) (Figure 3).

**TABLE 2:** Clinical outcomes of a duckbill‐type anti‐reflux self‐expandable metal stent (D‐ARMS)

	*n* (%)
Technical success	10 (100)
Functional success	10 (100)
Recurrent biliary obstruction	2 (20)
Occlusion	2 (20)
Overgrowth	1 (10)
Sludge formation	1 (10)
Migration	0 (0)
Adverse events other than recurrent biliary obstruction	
Early adverse events	1 (10)
Cholecystitis	1 (10)
Late adverse events	0 (0)

*n* = 10.

Abbreviation: D‐ARMS, duckbill‐type anti‐reflux self‐expandable metal stent.

After D‐ARMS placement, duodenal stent insertion was performed in one patient and gastrojejunal bypass surgery was performed in 1 patient, but neither patient had complications of RBO.

As an adverse event other than RBO, one case of cholecystitis developed early (at 15 days) after D‐ARMS placement due to an impacted gallstone in the cystic duct. Fluoroscopy confirmed that D‐ARMS did not cover the cystic duct. Although the patient improved following percutaneous drainage, there was repeated relapse. EUS‐guided gallbladder drainage was performed and the patient's condition improved.

## DISCUSSION

ARMS was developed for the purpose of prolonging TRBO and preventing duodenal fluid reflux, and various types of ARMS have been reported.^15^, ^16^, ^17^, ^18^, ^19^, ^20^, ^21^, ^22^ Randomized controlled trials that compared ARMS with conventional SEMS have also been conducted. Some reported that ARMS contributed to the extension of TRBO,^19^, ^20^, ^21^ whereas others reported no difference.^22^ It is considered that the disagreement in findings is largely due to differences in the shape and length of the anti‐reflux valve among ARMS; however, the optimum shape has not yet been determined. The D‐ARMS used in this study is shaped like a duck's bill, and the length of the check valve is relatively long (12.5 mm).

In the present study, we examined the usefulness and safety of D‐ARMS in the situation of duodenal invasion where duodenal fluid easily flowed back into the bile duct. We found no cases of non‐occlusion cholangitis within the observation period. Although it has been reported that migration is greater with ARMS than conventional SEMS, perhaps due to the high outflow pressure at the check valve,^22^, ^25^ no migration occurred in this study, which indicates the potential of D‐ARMS as an optimal ARMS. The shape of the check valve of the D‐ARMS and the fact that it is a laser‐cut type SEMS may have contributed to the lack of migration.

Recent reports from Japan have described the usefulness of this stent.^26^, ^27^, ^28^, ^29^, ^30^ In the one case of RBO caused by sludge formation in the present study, the check valve had collapsed. Kin et al reported RBO due to sludge formation in five patients, of whom the check valve had collapsed in two.^27^ Yamada et al reported RBO caused by sludge formation or non‐occlusion cholangitis in five cases, and the check valve had collapsed in all five.^30^ As it is expected that failure of the check valve will cause sludge formation and subsequent RBO, it is desirable to improve the durability of the check valve in the future. The fact that RBO due to sludge formation is likely to occur if there is a collapse of the anti‐reflux valve suggests that preventing the reflux of duodenal fluid would contribute to the prolongation of TRBO. It is thought that sludge formation is likely to occur if another stent is placed first, due to reflux of duodenal fluid into the bile duct. The authors of another study also concluded that laser‐cut type‐covered SEMS is the first choice for patients with unresectable distal malignant biliary obstruction.^31^ We consider that placing D‐ARMS, which is a laser‐cut type of covered SEMS, as the first stent may lead to suppression of sludge formation and eventually suppression of RBO.

For reintervention after RBO, it is desirable to place a new stent after removing the initial stent. It is easy to place a laser‐cut type‐covered SEMS at the target location, but removal is often difficult due to the characteristics of the stent. D‐AMRS removal was attempted in two of the present patients with RBO but was difficult, so we performed additional placement of SEMS in the form of a stent‐in‐stent. However, Kin et al. reported that D‐ARMS could be removed in six of nine cases that developed RBO,^27^ and Yamada et al reported that D‐ARMS could be removed in all six cases that developed RBO.^30^ Other studies also consider that laser‐cut type‐covered SEMS can be removed.^32^, ^33^ Whether or not D‐ARMS can be removed is controversial, and it is necessary to continue to accumulate cases in the future.

Early obstruction of a SEMS is more likely to occur in the presence of duodenal invasion,^9^, ^10^, ^11^, ^12^, ^13^, ^14^ which suggests that EUS‐guided drainage may be more useful than transpapillary drainage.^34^, ^35^ However, EUS‐guided drainage is difficult in patients with a large amount of ascites or without bile duct dilatation. In addition, EUS‐guided drainage is a relatively new procedure that is not yet widespread, and there are limited facilities where this treatment is possible. In contrast, transpapillary drainage using D‐ARMS can be performed regardless of ascites or bile duct dilation, and because it is a standard ERCP procedure, it can be performed at many facilities. Therefore, transpapillary drainage using D‐ARMS is more universal than EUS‐guided drainage and is widely accessible.

There are several limitations to this study, including its small sample size, single‐arm retrospective study design, and short observation period. In addition, the intervention was performed for duodenal tumor invasion in only two patients. As we included patients in whom the mucosal layer was visually invaded by endoscopy as well as those in whom only the duodenal muscular layer was invaded by EUS, the gastric outlet obstruction symptoms might not have developed within the observation period. In the pathological evaluation of surgical specimens, duodenal invasion is defined as invasion of the muscular layer even if the mucosal layer of the duodenum is not invaded. In the past, the duodenal invasion was defined as the endoscopic observation of invasion into the mucosal layer. Now that the use of EUS is widespread, we think that the definition of duodenal invasion should include invasion into the muscle layer on EUS. If the invasion is observed in the muscular layer, duodenal peristalsis is reduced, and it is thought that reflux of duodenal fluid into the bile duct is likely to occur. The large number of patients who died relatively early after stent placement affected the follow‐up data. Further verification in randomized controlled trials is required in the future.

## CONFLICTS OF INTEREST

The authors declare that they have no conflict of interest.

## FUNDING INFORMATION

This research received no external funding.

## ETHICS STATEMENT

The study was conducted according to the guidelines of the Declaration of Helsinki and approved by the Institutional Review Board of Dokkyo Medical University Saitama Medical Center (approval no.: 21036; approval date: 4 June 2021).
